# Crystal structure of {μ_2_-1,2-bis­[(4-methyl­phenyl­sulfan­yl]-3-oxoprop-1-ene-1,3-di­yl-1:2κ^2^ *C* ^3^:*C* ^1^}dicarbon­yl-1κ^2^ *C*-[μ_2_-methyl­enebis(di­phenyl­phos­phane)-1:2κ^2^ *P*:*P*′](tri­phenyl­phosphane-2κ*P*)iron­platinum(*Fe*—*Pt*), [(OC)_2_Fe(μ-dppm){μ-C(=O)C(4-MeC_6_H_4_SCH_2_)=CCH_2_SC_6_H_4_Me-4}Pt(PPh_3_)]

**DOI:** 10.1107/S2056989020007859

**Published:** 2020-06-16

**Authors:** Ahmed Said Mohamed, Isabelle Jourdain, Michael Knorr, Lukas Brieger, Carsten Strohmann

**Affiliations:** a Centre D’Etude et de Recherche de Djibouti, Djibouti; b Institut UTINAM UMR 6213 CNRS, Université Bourgogne Franche-Comté, 16 Route de Gray, 25030 Besançon, France; c Institute for Inorganic Chemistry, Faculty of Chemistry and Chemical Biology, TU Dortmund University, Otto-Hahn Str. 6, 44227 Dortmund, Germany

**Keywords:** crystal structure, inter­nal alkyne, iron, platinum, heterobimetallic, metal–metal bond, dimetallcyclo­pentenone, bis­(di­phenyl­phosphino)methane, thio­ether, hydrogen bonding

## Abstract

The title compound, [FePt(C_19_H_18_OS_2_)(C_18_H_15_P)(C_25_H_22_P_2_)(CO)_2_], **1**, [(OC)_2_Fe(μ-dppm)(μ-C(=O)C(CH_2_SC_6_H_4_Me-4)=CCH_2_SC_6_H_4_Me-4)Pt(PPh_3_)], represents the first example of a diphosphane-bridged heterobimetallic Fe—Pt dimetalla­cyclo­pentenone complex resulting from a bimetallic activation of metal-coordinated carbonyl ligand with an inter­nal alkyne, namely 1,4-bis­(*p*-tolyl­thio)­but-2-yne. The bridging μ_2_-C(=O)C(CH_2_SC_6_H_4_Me-4)=CCH_2_SC_6_H_4_Me-4 unit (stemming from a carbon–carbon coupling reaction between CO and the triple bond of the alkyne di­thio­ether) forms a five-membered dimetalla­cyclo­pentenone ring, in which the C=C bond is π-coordinated to the Fe center. The latter is connected to the Pt center through a short metal–metal bond of 2.5697 (6) Å.

## Abstract 2

The title compound represents the first example of a diphosphane-bridged heterobimetallic Fe—Pt dimetalla­cyclo­pentenone complex resulting from a bimetallic activation of metal-coordinated carbonyl ligand with an inter­nal alkyne, namely 1,4-bis­(*p*-tolyl­thio)­but-2-yne. The bridging μ_2_-C(= O)C(CH_2_SC_6_H_4_Me-4)=CCH_2_SC_6_H_4_Me-4 unit (stemming from a carbon–carbon coupling reaction between CO and the triple bond of the alkyne di­thio­ether) forms a five-membered dimetalla­cyclo­pentenone ring, in which the C=C bond is π-coordinated to the Fe center.

## Chemical context   

**Figure 1:**
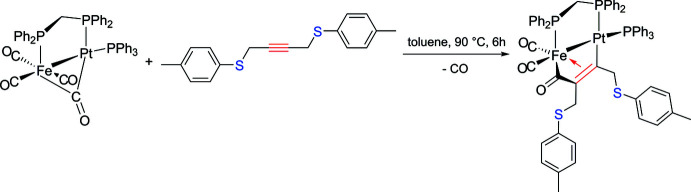
The reaction scheme for the synthesis of **1**.

Acetyl­enic di­thio­ether ligands of type *R*SCH_2_C≡CCH_2_S*R* (*R* = aryl, alk­yl) have in recent years not only attracted attention as reactive building blocks for further organic transformations (Pourcelot & Cadiot, 1966 ▸; Everhardus & Brandsma; 1978 ▸; Levanova *et al.*, 2015 ▸) but also as promising ligands for coordination chemistry because of their dytopic character, allowing both coordination to soft metal centers through dative *M*←S bonding and π-bonding *via* the acetyl­enic triple bond. In this context, we have explored in a series of several papers the coordination of this ligand family to Cu*X* salts in a self-assembly process to discrete mol­ecular compounds, mono- and bidimensional coordination polymers and three-dimensional MOFs. For example, treatment of CuI with PhSCH_2_C≡CCH_2_SPh afforded a three-dimensional network incorporating Cu_6_(μ_3_-I) hexa­gonal prisms as connection nodes (Knorr *et al.*, 2009 ▸; Bai *et al.*, 2018 ▸). In contrast, reaction of BzSCH_2_C≡CCH_2_SBz (Bz = benz­yl) with both CuI and CuBr provided simple isostructural dinuclear zero-dimensional complexes [{Cu(μ_2_-*X*)_2_Cu}(μ-BzSCH_2_C≡CCH_2_SBz)_2_] (*X* = I, Br). A far more original material resulted from coordination to CuCl, yielding a luminescent 2D material [{Cu_2_(μ_2_-Cl)(μ_3_-Cl)}(μ-BzSCH_2_C≡CCH_2_SBz)]_*n*_, in which the layers are assembled both by dative Cu—S thio­ether bonds and organometallic Cu-π–acetyl­enic inter­actions *via* the triple bond of the ligand. Furthermore, the Cu^I^ centers are inter­connected through μ_2_- and μ_3_-bound chloro ligands. Treatment of CuI with the isomeric *p*-TolSCH_2_C≡CCH_2_STol-*p* (Tol = C_6_H_4_-*p*-Me) ligand led to the formation of a 2D network [{Cu_4_(μ_3_-I)_4_}(μ-TolSCH_2_C≡CCH_2_STol)_2_]_*n*_ with closed cubane-type clusters as SBUs (Secondary Building Units), whilst with CuBr the 1D [{Cu(μ_2_-Br)_2_Cu}(μ-TolSCH_2_C≡CCH_2_STol)_2_]_*n*_ coordination polymer was generated (Aly *et al.*, 2014 ▸; Bonnot *et al.*, 2015 ▸). An alternative approach to combining a metallic scaffold with *R*SCH_2_C≡CCH_2_S*R*-type ligands has been developed by Went and coworkers, who post-functionalized dicobalta­tetra­hedrane complexes [Co_2_(μ-HOCH_2_C≡CCH_2_OH)(CO)_6_] in the presence of HBF_4_·OEt_2_ and various thiols *R*SH to obtain [Co_2_(μ-*R*SCH_2_C≡CCH_2_S*R*)(CO)_6_] and [Co_2_(μ-*R*SCH_2_C≡CCH_2_S*R*)(μ-dppm)(CO)_4_] [dppm = bis­(di­phenyl­phosphino)methane], respectively. Similar treatment of [Mo_2_(μ-HOCH_2_C≡CCH_2_OH)(CO)_4_Cp_2_] with EtSH yielded [Mo_2_(μ-EtSCH_2_C≡CCH_2_SEt)(CO)_4_Cp_2_]. These former Co–Co thio­ether complexes were then employed as metalloligands to coordinate further metal fragments such as [Cu(MeCN)_4_]PF_6_, AgBF_4_ and [Mo(CO)_4_(norbornadiene)] (Bennett, *et al.*, 1992 ▸; Gelling *et al.*, 1993 ▸). Related dicationic salts such as [(Co_2_(CO)_6_)_2_-μ,η^2^,η^2^-(Me_2_S—CH_2_C≡CCH_2_SMe_2_)][BF_4_]_2_ have also been described (Amouri *et al.*, 2000 ▸). We and Shaw’s group have demonstrated that upon treatment of the μ-carbonyl complex [(OC)_3_Fe(μ-dppm)(μ-CO)Pt(PPh_3_)] with ArC≡CH (Ar = Ph, *p*-Tol, 2,4,5-tri­methyl­phenyl, *p*-C_6_H_4_F, 2,4-C_6_H_3_F_2_, *p*-C_6_H_4_CF_3_), dimetalla­cyclo­pentone complexes are formed, stemming from carbon–carbon coupling reactions between CO and the terminal alkyne (Fontaine *et al.*, 1988 ▸; Jourdain *et al.*, 2013 ▸; Knorr & Jourdain, 2017 ▸; Brieger *et al.*, 2019 ▸). The first step involves the formation of a kinetic isomer [(OC)_2_Fe(μ-dppm){μ-C(=O)C(H)=C(Ar)}Pt(PPh_3_)], which then evolves to the thermodynamic one [(OC)_2_Fe(μ-dppm){μ-C(=O)C(Ar)=C(H)}Pt(PPh_3_)]. We were now intrigued as to whether this route may be extended to inter­nal alkynes *R*C≡C*R*, which are in general less reactive than terminal ones. We therefore probed the possibility of coupling [(OC)_3_Fe(μ-dppm)(μ-CO)Pt(PPh_3_)] with *p*-TolSCH_2_C≡CCH_2_STol-*p* in hot toluene as solvent and succeeded in isolating the targeted dimetalla­cyclo­pentone [(OC)_2_Fe(μ-dppm)(μ-C(=O)C(4*-*MeC_6_H_4_SCH_2_)=CCH_2_SC_6_H_4_Me-4)Pt(PPh_3_)] (**1**) as a stable crystalline product according to the reaction scheme shown in Fig. 1 ▸. With this title compound **1** in hand, we now have the possibility of coordinating other metal fragments in upcoming studies, for example [Mo(CO)_4_(norbornadiene)] or ReBr(CO)_5_ in a chelating manner using the two adjacent thio­ether arms or of constructing coordination networks incorporating complex **1** as an organometallic building block by coordination of Cu*X* or Ag^I^ salts on the *S*-donor sites (see above).
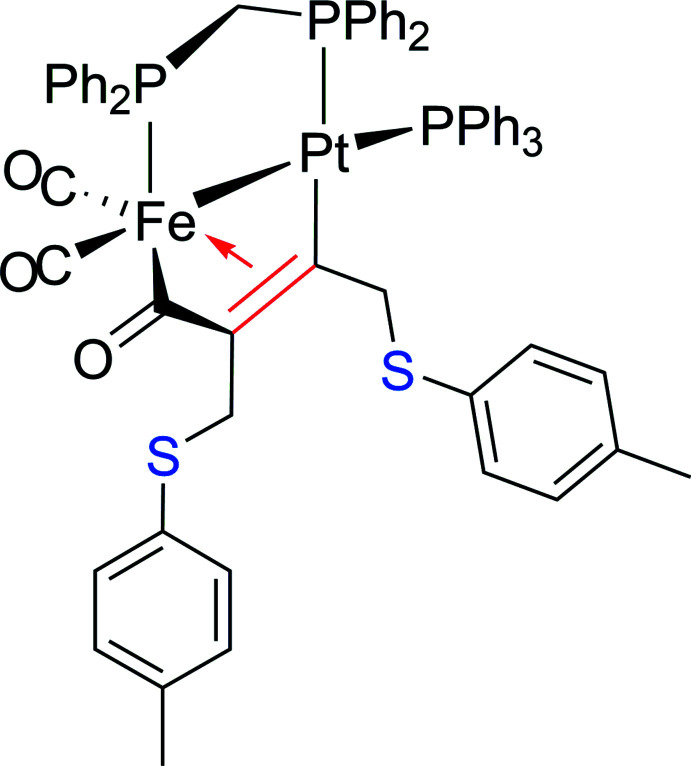


## Structural commentary   

**Figure 2:**
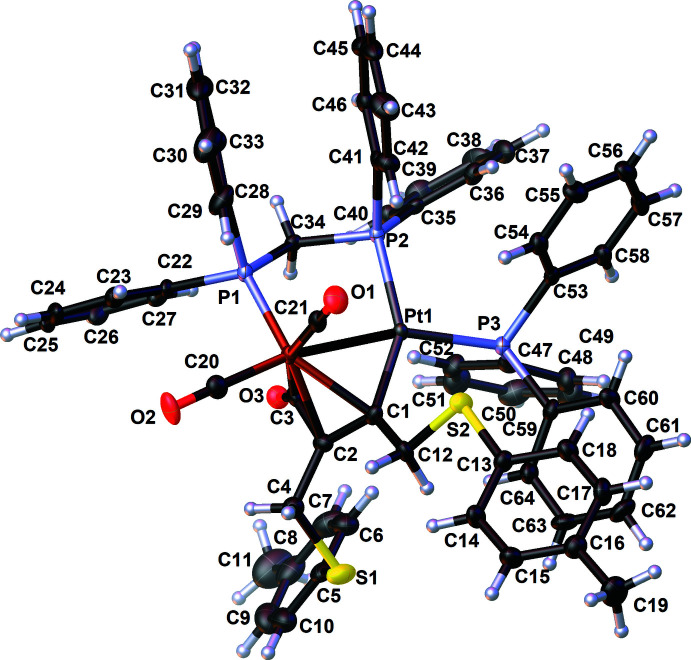
The mol­ecular structure of the title complex **1**, with atom labeling. Displacement ellipsoids are drawn at the 30% probability level.

The heterobimetallic compound **1** crystallizes in the monoclinic crystal system, space group *P*2_1_/*c*. The mol­ecular structure is depicted in Fig. 2 ▸ and selected bond lengths and angles are given in Table 1 ▸.

The Fe—Pt bond [2.5697 (6) Å] is spanned by a dppm ligand and bridged by the C(=O)C(*R*)=C(*R*) (*R* = 4*-*MeC_6_H_4_SCH_2_) unit resulting from the carbon–carbon coupling reaction between CO and the alkyne. This value, which is less than 2.6 Å, is in the usual range for FePt(dppm)–dimetalla­cyclo­pentenone complexes. Note that extreme Fe—Pt distances are reported for the μ-carbene [(OC)_3_Fe{μ-C(Et)OSi(OMe)_3_}(μ-dppm)Pt(PPh_3_)] [*d*(Fe—Pt) = 2.5062 (9) Å; YOTCIT; Braunstein *et al.*, 1995 ▸] and [Fe(η^5^-C_5_H_4_S)_2_Pt(PPh_3_)] [*d*(Fe—Pt = 2.935 (2) Å; FENCUW; Akabori *et al.*, 1987 ▸]. Coupling of an inter­nal alkyne does not affect the structural features of the [FeC(=*O*)C(*R*)=C(*R*)Pt] motif significantly with respect to carbon–carbon coupling with a terminal alkyne. The relevant bond lengths and angles are very similar to those of other Fe—Pt structures published by Fontaine *et al.* (1988 ▸) and our group (see above). The presence of a bulky substituent on the C1 atom bound to platinum implies a significant reduction of the P3—Pt—P2 angle [100.53 (4)°] concomitant with an increasing value of the angle P3—Pt—C1 of 107.33 (12)°. In related compounds described previously in the literature, these P3—Pt—P2 angles usually lie in the range 103.93 (8) to 106.63 (3)°, as exemplified by [(OC)_2_Fe(μ-dppm){μ-C(=O)C{(CH_2_)_3_CCH}=C(H)}Pt(PPh_3_)] (REDNEU) and [(OC)_2_Fe(μ-dppm){μ-C(=O)C(*p*-C_6_H_4_CF_3_)=C(H)}Pt(PPh_3_)] (PIXLAL), and 98.8 (3) to 104.95 (10)° for P3—Pt—C1 in [(OC)_2_Fe(μ-dppm){μ-C(=O)C(H)=C(H)}Pt(PPh_3_)] (FEYBAM) and [(OC)_2_Fe(μ-dppm){μ-C(=O)C(*o,p*-C_6_H_3_F_2_)=C(H)}Pt(PPh_3_)] (PIX­KUE) (Fontaine *et al.*, 1988 ▸; Jourdain *et al.*, 2006 ▸, 2013 ▸). The crystal structure of the di­thio­ether *p*-TolSCH_2_C≡CCH_2_STol-*p* (MULHUZ) was reported by Aly *et al.* (2014 ▸). After complexation and a coupling reaction with a CO ligand, the C1—C2 bond is considerably longer [1.407 (6) *vs* 1.266 (5) Å] as a result of the conversion to an olefinic moiety, σ-bound to Pt and η^2^-coordinated to Fe. The alkyne bending angles are disparate [C1—C2—C4 = 126.2 (4), C2—C1—C12 = 119.6 (4)°] as well as the C1—C12 and C2—C4 distances [*d(*C1—C12) = 1.483 (6), *d*(C2—C4) = 1.511 (5) Å]. Compared to 1,4-bis­(*p*-tolyl­thio)­but-2-yne, the C—S bonds are also considerably elongated [*d*(C4—S1 = 1.830 (4), *d*(C12—S2) = 1.808 (4), *d*(C5—S1) = 1.782 (5), *d*(C13—S2) = 1.771 (4) *vs* 1.685 (2) and 1.714 (2) Å] but they fit well with those encountered in the dimetalla­tetra­hedrane [Co_2_{μ-C_2_(CH_2_SMe)_2_Mo(CO)_4_}(μ-dppm)(CO)_4_] [*d*(C—S = 1.827 (4), 1.833 (4),1.790 (5) and 1.819 (5) Å; JIHMUI10; Gelling *et al.*, 1993 ▸].

## Supra­molecular features   

**Figure 3:**
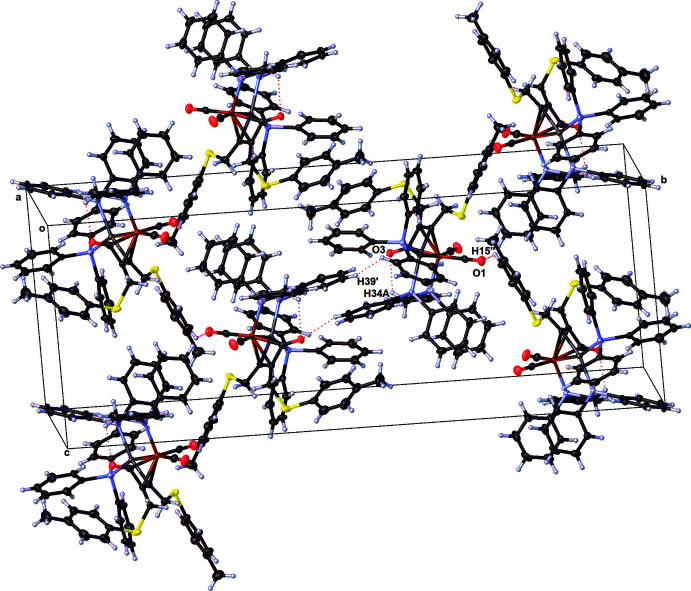
A partial view along the *a* axis of the crystal packing of the title compound. The hydrogen bonds (Table 2 ▸) are shown as dashed lines.

In the crystal, the individual mol­ecules are linked by weak inter­molecular inter­actions; for example a contact between O3′′⋯H39 [*d* = 2.49 Å and C3′′—O3′′⋯H39 = 138°; symmetry code: (′′) −*x* + 1, −*y* + 1, −*z* + 1] occurs (Fig. 3 ▸, Table 2 ▸. A second, yet still weaker inter­molecular inter­action of 2.67 Å is observed between the O1′⋯H15 [symmetry code: (′) *x*, –*y* + 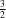, *z* − 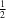] atoms of two adjacent mol­ecules. In addition there is an intra­molecular contact between O3⋯H34*A* (*d* = 2.62 Å and C3—O3⋯H34*A* = 125°). Furthermore, there are also several loose inter­molecular C—H⋯π inter­actions present; for example a contact between C43—H43 and the midpoint of the C13=C14 double bond [*d*(H43⋯midpoint) = 2.73 Å and C—H⋯midpoint = 157°] of a tolyl ring attached to S2, as well as between C62—H62 and the C23—C24—C25 atoms of a phenyl ring [*d*(H62⋯centroid) = 2.64 Å and C—H⋯centroid = 148°] attached at P1. However, since all hydrogen atoms were not refined freely, a more accurate discussion of the bond lengths and angle is not appropriate.

## Database survey   

Other examples of crystallographically characterized dimetalla­cyclo­pentenone complexes are Fe_2_Cp_2_(CO)(μ-CO){μ-CH=C(Ph)C(=O)} (DAHTAJ; Boni *et al.*, 2011 ▸), Fe_2_Cp*_2_(CO)(μ-CO){μ-C(C≡CH)=CHC(=O)] (JUZHIV; Akita *et al.*, 1993 ▸), Fe_2_(CO)_5_(μ-dppm){μ-C(=O)CH=CH} (GACWIQ10; Knox *et al.*, 1995 ▸), Fe_2_(CO)_5_(μ-dppm){μ-C(=O)C(Ph)=CH} (PIHMOI; Hitchcock *et al.*, 1993 ▸), Fe_2_Cp_2_(CO)(μ-CO){μ-C(CO*R*)=C(Me)C(=O)} (*R* = Ph, Bu) (SIZNUK, SIZPAS; Wong *et al.*, 1991 ▸), Fe_2_{(η-C_5_H_4_)_2_SiMe_2_}(CO)_2_(μ-CO){μ-C(Ph)=C(H)C(=O)} (ZUZGIK; McKee *et al.*, 1994 ▸), Ru_2_(CO)_4_(μ-dppm)_2_{μ-C(=O)C(CO_2_Me)=C(CO_2_Me)} (JITZAN; Johnson & Gladfelter, 1991 ▸), Ru_2_(CO)_4_(μ-dppm)_2_{μ-CH=CHC(=O)} (LIFYUU; Mirza *et al.*, 1994 ▸), Ru_2_(η-C_5_HMe_4_)_2_(CO)(μ-CO){μ-C(=O)C(*R*)=C(*R*)} (*R* = Et, Me) (NEMVOS, NEMVUY; Horiuchi *et al.*, 2012 ▸), Rh_2_Cp_2_(CO)_4_{μ-C(CF_3_)=C(CF_3_)C(=O)} (TFPNRH; Dickson *et al.*, 1981 ▸), Re_2_Cp*_2_(CO)_2_{μ-CH=C{C(=CH_2_)CH_3_}C(=O)} (WEZKIV; Casey *et al.*, 1994 ▸). A rare example of a heterodinuclear combination is CpFe{μ-C(=O)C(CMe_2_OH)=CH}(μ-CO)Ru(CO)Cp* (FEHGOP; Dennett *et al.*, 2005 ▸). We are also aware of OsRu(CO)_8_{μ-HC=CHC(=O)} (Kiel *et al.*, 2000 ▸), but for the latter compound no structural data are available.

## Synthesis and crystallization   

[(OC)_3_Fe(μ-CO)(μ-dppm)Pt(PPh_3_)] (200 mg, 0.2 mmol) was treated with an excess of 1,4-bis­(*p*-tolyl­thio)­but-2-yne (100 mg, 0.4 mmol) in toluene (5 mL). The solution was stirred at 363 K for 6h. The reaction mixture was filtered, and all volatiles removed under reduced pressure. The brown residue was redissolved in a minimum of toluene. Orange–yellow crystals were isolated by layering with heptane (152 mg, 76% yield).

Calculated for C_64_H_55_FeO_3_P_3_PtS_2_ (1279.18 g mol^−1^): C, 60.05; H, 4.36. Found: C, 59.80; H, 4.21. ^1^H NMR: δ 2.21 (*s*, 3H, CH_3_), 2.28 (*s*, 3H, CH_3_), 3.67 (*br*, 2H, CH_2_), 3.97(*br*, 2H, CH_2_), 4.53 (*br*, 2H, PCH_2_P, ^2^ *J*_PtH_ = 41), 6.45–7.85 (*m*, 43H, Ph). ^31^P{1H} NMR: δ 6.8 (*d*, P_dppm Pt_, ^2^ *J*_PP_ = 57, ^2 + 3^ *J*_PP_ = 5, ^1^ *J*_PtP_ = 2543), 32.7 (*d*, P_PPh3 Pt_, ^3^ *J*_PP_ = 32, ^2 + 3^ *J*_PP_ = 5, ^1^ *J*_PtP_ = 3506), 63.4 (*dd*, P_dppm Fe_, ^2^ *J*_PP_ = 57, ^3^ *J*_PP_ = 32, ^1^ *J*_PtP_ = 135). IR(toluene): 1966, 1918s ν(CO), 1696m ν(C=O).

## Refinement   

Crystal data, data collection and structure refinement details are summarized in Table 3 ▸. All of the hydrogen atoms were placed in geometrically calculated positions and each was assigned a fixed isotropic displacement parameter based on a riding model: C—H = 0.93–0.97 Å with *U*_iso_(H) = 1.5*U*_eq_(C-meth­yl) and 1.2*U*_eq_(C) for other H atoms.

## Supplementary Material

Crystal structure: contains datablock(s) I. DOI: 10.1107/S2056989020007859/pk2630sup1.cif

Structure factors: contains datablock(s) I. DOI: 10.1107/S2056989020007859/pk2630Isup3.hkl

CCDC reference: 1996804

Additional supporting information:  crystallographic information; 3D view; checkCIF report

## Figures and Tables

**Table 1:** Selected geometric parameters (Å, °)

Pt1—Fe1	2.5697 (6)	Fe1—C2	2.119 (4)
Pt1—P2	2.2850 (10)	Fe1—C3	1.932 (5)
Pt1—P3	2.2714 (12)	Fe1—C20	1.777 (5)
Pt1—C1	2.045 (4)	Fe1—C21	1.789 (5)
Fe1—P1	2.1966 (12)	O3—C3	1.216 (5)
Fe1—C1	2.162 (4)	C1—C2	1.407 (6)
			
P2—Pt1—Fe1	97.26 (3)	C1—Fe1—C2	38.35 (16)
P3—Pt1—Fe1	161.46 (3)	C20—Fe1—P1	95.66 (14)
P3—Pt1—P2	100.53 (4)	C21—Fe1—P1	102.63 (13)
C1—Pt1—Fe1	54.44 (12)	C3—Fe1—P1	88.88 (13)
C1—Pt1—P2	151.36 (12)	C1—Fe1—P1	141.85 (12)
C1—Pt1—P3	107.33 (12)	C2—Fe1—P1	130.91 (13)
Pt1—C1—Fe1	75.25 (13)	C20—Fe1—Pt1	168.78 (14)
C20—Fe1—C3	101.5 (2)	C21—Fe1—Pt1	87.76 (14)
C20—Fe1—C21	96.5 (2)	C2—Fe1—Pt1	73.72 (12)
C21—Fe1—C3	157.6 (2)	C1—Fe1—Pt1	50.30 (11)
C20—Fe1—C1	119.19 (18)	C3—Fe1—Pt1	72.18 (13)
C21—Fe1—C1	89.16 (18)	P1—Fe1—Pt1	93.50 (4)
C3—Fe1—C1	70.43 (18)	C2—C1—Pt1	109.1 (3)
C20—Fe1—C2	95.33 (18)	C2—C1—Fe1	69.2 (2)

**Table 2:** Hydrogen-bond geometry (Å, °)

*D*—H⋯*A*	*D*—H	H⋯*A*	*D*⋯*A*	*D*—H⋯*A*
C15—H15⋯O1^i^	0.93	2.67	3.316 (6)	128
C34—H34*A*⋯O3	0.97	2.62	3.271 (5)	125
C39—H39⋯O3^ii^	0.93	2.49	3.239 (6)	138

Symmetry codes: (i) 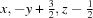; (ii) 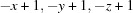.

**Table 3:** Experimental details

Crystal data	
Chemical formula	[FePt(C_19_H_18_OS_2_)(C_18_H_15_P)(C_25_H_22_P_2_)(CO)_2_]
*M*_r_	1280.05
Crystal system, space group	Monoclinic, *P*2_1_/*c*
Temperature (K)	293
*a*, *b*, *c* (Å)	12.0071 (6), 36.1737 (15), 13.6980 (6)
β (°)	111.970 (5)
*V* (Å^3^)	5517.5 (5)
*Z*	4
Radiation type	Mo *K*α
μ (mm^−1^)	3.01
Crystal size (mm)	0.23 × 0.15 × 0.05

Data collection	
Diffractometer	Agilent Technologies Xcalibur, Sapphire3
Absorption correction	Multi-scan (*CrysAlis PRO*; Agilent, 2014 ▸)
*T*_min_, *T*_max_	0.837, 1.000
No. of measured, independent and observed [*I* > 2σ(*I*)] reflections	47863, 10566, 8245
*R*_int_	0.071
(sin θ/λ)_max_ (Å^−1^)	0.611

Refinement	
*R*[*F* ^2^ > 2σ(*F* ^2^)], *wR*(*F* ^2^), *S*	0.037, 0.076, 1.03
No. of reflections	10566
No. of parameters	669
H-atom treatment	H-atom parameters constrained
Δρ_max_, Δρ_min_ (e Å^−3^)	1.12, −0.64

Computer programs: *CrysAlis PRO* (Agilent, 2014 ▸), *SHELXT2014/5* (Sheldrick, 2015*a* ▸), *SHELXL* (Sheldrick, 2015*b* ▸) and *OLEX2* (Dolomanov *et al.*, 2009 ▸).

## supplementary crystallographic information

### Crystal data

**Table:**

[FePt(C_19_H_18_OS_2_)(C_18_H_15_P)(C_25_H_22_P_2_)(CO)_2_]	*F*(000) = 2576
*M**_r_* = 1280.05	*D*_x_ = 1.541 Mg m^−^^3^
Monoclinic, *P*2_1_/*c*	Mo *K*α radiation, λ = 0.71073 Å
*a* = 12.0071 (6) Å	Cell parameters from 9511 reflections
*b* = 36.1737 (15) Å	θ = 2.7–28.7°
*c* = 13.6980 (6) Å	µ = 3.01 mm^−^^1^
β = 111.970 (5)°	*T* = 293 K
*V* = 5517.5 (5) Å^3^	Plate, yellow
*Z* = 4	0.23 × 0.15 × 0.05 mm

### Data collection

**Table:**

Agilent Technologies Xcalibur, Sapphire3 diffractometer	10566 independent reflections
Radiation source: Enhance (Mo) X-ray Source	8245 reflections with *I* > 2σ(*I*)
Graphite monochromator	*R*_int_ = 0.071
Detector resolution: 16.0560 pixels mm^-1^	θ_max_ = 25.8°, θ_min_ = 2.2°
ω scans	*h* = −11→14
Absorption correction: multi-scan (CrysAlisPro; Agilent, 2014)	*k* = −44→41
*T*_min_ = 0.837, *T*_max_ = 1.000	*l* = −16→16
47863 measured reflections	

### Refinement

**Table:**

Refinement on *F*^2^	Primary atom site location: dual
Least-squares matrix: full	Hydrogen site location: inferred from neighbouring sites
*R*[*F*^2^ > 2σ(*F*^2^)] = 0.037	H-atom parameters constrained
*wR*(*F*^2^) = 0.076	*w* = 1/[σ^2^(*F*_o_^2^) + (0.0274*P*)^2^ + 0.5475*P*] where *P* = (*F*_o_^2^ + 2*F*_c_^2^)/3
*S* = 1.03	(Δ/σ)_max_ = 0.005
10566 reflections	Δρ_max_ = 1.12 e Å^−^^3^
669 parameters	Δρ_min_ = −0.64 e Å^−^^3^
0 restraints	

### Special details

**Table:**

Geometry. All esds (except the esd in the dihedral angle between two l.s. planes) are estimated using the full covariance matrix. The cell esds are taken into account individually in the estimation of esds in distances, angles and torsion angles; correlations between esds in cell parameters are only used when they are defined by crystal symmetry. An approximate (isotropic) treatment of cell esds is used for estimating esds involving l.s. planes.
Refinement. Crystal structure determination of compound 1 was accomplished on an Oxford diffraction Xcalibur S Diffractometer. Suitable crystals of 1 were covered with an inert oil (perfluoropolyalkylether and used for X-ray crystal structure determination. Graphite monochromated Mo-Kα radiation (λ = 0.71073?Å) was used. The processing and finalization of the crystal structure was done with the program Olex2 (Dolomanov, 2009). The crystal structures were solved by intrinsic phasing (SHELXT; Sheldrick, 2015a) and refined against F2 with the full-matrix least-squares method (SHELXL; Sheldrick, 2015b). A multi-scan absorption correction using the CrysAlis RED program (Oxford Diffraction, 2010) was employed. The non-hydrogen atoms were refined anisotropically.

### Fractional atomic coordinates and isotropic or equivalent isotropic displacement parameters (Å^2^)

**Table:**

	*x*	*y*	*z*	*U*_iso_*/*U*_eq_	
Pt1	0.33424 (2)	0.62472 (2)	0.31155 (2)	0.01386 (6)	
Fe1	0.52875 (5)	0.66228 (2)	0.35654 (4)	0.01541 (14)	
S1	0.51458 (12)	0.61606 (4)	0.02018 (9)	0.0335 (3)	
S2	0.19884 (11)	0.69548 (4)	0.12684 (8)	0.0242 (3)	
P1	0.60180 (10)	0.64353 (3)	0.52099 (8)	0.0160 (3)	
P2	0.36370 (10)	0.60649 (3)	0.47912 (8)	0.0136 (2)	
P3	0.16428 (10)	0.59290 (3)	0.21914 (8)	0.0166 (3)	
O1	0.3898 (3)	0.72834 (9)	0.3663 (2)	0.0305 (8)	
O2	0.7452 (3)	0.70095 (9)	0.3644 (3)	0.0331 (8)	
O3	0.6059 (3)	0.58365 (9)	0.3412 (2)	0.0226 (7)	
C1	0.3927 (4)	0.64918 (12)	0.2048 (3)	0.0167 (10)	
C2	0.5040 (4)	0.63378 (12)	0.2148 (3)	0.0184 (10)	
C3	0.5576 (4)	0.61346 (14)	0.3138 (3)	0.0199 (11)	
C4	0.5728 (4)	0.64158 (13)	0.1445 (3)	0.0214 (10)	
H4A	0.656425	0.635059	0.181663	0.026*	
H4B	0.569106	0.667854	0.129478	0.026*	
C5	0.5750 (4)	0.57103 (14)	0.0594 (3)	0.0284 (12)	
C6	0.5322 (5)	0.54791 (15)	0.1179 (4)	0.0356 (13)	
H6	0.469522	0.555719	0.137208	0.043*	
C7	0.5815 (5)	0.51369 (15)	0.1475 (4)	0.0413 (15)	
H7	0.552057	0.498782	0.187748	0.050*	
C8	0.6741 (5)	0.50038 (15)	0.1194 (4)	0.0398 (14)	
C9	0.7145 (5)	0.52324 (16)	0.0597 (4)	0.0436 (15)	
H9	0.775600	0.514984	0.038988	0.052*	
C10	0.6673 (5)	0.55811 (16)	0.0295 (4)	0.0364 (14)	
H10	0.696839	0.572966	−0.010742	0.044*	
C11	0.7276 (6)	0.46250 (16)	0.1544 (5)	0.065 (2)	
H11A	0.743383	0.459346	0.227930	0.097*	
H11B	0.801292	0.460237	0.142632	0.097*	
H11C	0.672116	0.443892	0.114692	0.097*	
C12	0.3296 (4)	0.67416 (13)	0.1153 (3)	0.0208 (11)	
H12A	0.305131	0.660195	0.050210	0.025*	
H12B	0.384620	0.693322	0.112025	0.025*	
C13	0.1237 (4)	0.71380 (13)	−0.0009 (3)	0.0213 (11)	
C14	0.1814 (4)	0.72895 (13)	−0.0630 (3)	0.0256 (11)	
H14	0.264781	0.728200	−0.039538	0.031*	
C15	0.1159 (4)	0.74513 (14)	−0.1592 (3)	0.0299 (12)	
H15	0.156559	0.755564	−0.198448	0.036*	
C16	−0.0078 (4)	0.74615 (13)	−0.1983 (3)	0.0264 (11)	
C17	−0.0649 (4)	0.73051 (14)	−0.1363 (3)	0.0286 (12)	
H17	−0.148331	0.730799	−0.160551	0.034*	
C18	−0.0010 (4)	0.71470 (13)	−0.0406 (3)	0.0252 (11)	
H18	−0.041978	0.704399	−0.001440	0.030*	
C19	−0.0794 (5)	0.76391 (16)	−0.3034 (4)	0.0428 (15)	
H19A	−0.047412	0.787999	−0.306543	0.064*	
H19B	−0.161883	0.766129	−0.310870	0.064*	
H19C	−0.074281	0.748835	−0.359219	0.064*	
C20	0.6604 (4)	0.68613 (13)	0.3621 (3)	0.0230 (11)	
C21	0.4445 (4)	0.70239 (13)	0.3626 (3)	0.0199 (10)	
C22	0.7607 (4)	0.62972 (13)	0.5705 (3)	0.0176 (10)	
C23	0.8454 (4)	0.65829 (14)	0.5896 (3)	0.0246 (11)	
H23	0.820210	0.682797	0.581983	0.030*	
C24	0.9660 (4)	0.65016 (16)	0.6197 (3)	0.0326 (13)	
H24	1.021532	0.669251	0.632344	0.039*	
C25	1.0048 (4)	0.61401 (16)	0.6313 (3)	0.0324 (14)	
H25	1.085863	0.608652	0.649885	0.039*	
C26	0.9219 (4)	0.58565 (15)	0.6149 (3)	0.0315 (13)	
H26	0.947659	0.561191	0.624560	0.038*	
C27	0.8012 (4)	0.59378 (13)	0.5841 (3)	0.0234 (11)	
H27	0.746189	0.574587	0.572385	0.028*	
C28	0.6025 (4)	0.67405 (13)	0.6288 (3)	0.0198 (10)	
C29	0.5891 (5)	0.71186 (14)	0.6141 (4)	0.0324 (13)	
H29	0.581547	0.721995	0.549531	0.039*	
C30	0.5869 (5)	0.73480 (15)	0.6941 (4)	0.0423 (15)	
H30	0.577689	0.760181	0.683252	0.051*	
C31	0.5983 (5)	0.71993 (16)	0.7901 (4)	0.0358 (13)	
H31	0.595101	0.735182	0.843640	0.043*	
C32	0.6144 (5)	0.68300 (15)	0.8061 (4)	0.0344 (13)	
H32	0.623800	0.673087	0.871411	0.041*	
C33	0.6168 (4)	0.65998 (13)	0.7268 (3)	0.0250 (11)	
H33	0.628129	0.634720	0.739143	0.030*	
C34	0.5268 (4)	0.60132 (12)	0.5404 (3)	0.0150 (10)	
H34A	0.552067	0.580369	0.509554	0.018*	
H34B	0.549824	0.596640	0.615122	0.018*	
C35	0.3097 (4)	0.56202 (12)	0.5064 (3)	0.0160 (10)	
C36	0.1925 (4)	0.55998 (13)	0.5041 (3)	0.0218 (11)	
H36	0.144301	0.580982	0.488889	0.026*	
C37	0.1481 (5)	0.52697 (14)	0.5244 (3)	0.0289 (12)	
H37	0.069837	0.525755	0.522178	0.035*	
C38	0.2195 (5)	0.49550 (14)	0.5482 (4)	0.0326 (13)	
H38	0.189317	0.473274	0.561975	0.039*	
C39	0.3353 (5)	0.49742 (13)	0.5512 (3)	0.0283 (12)	
H39	0.383817	0.476506	0.567991	0.034*	
C40	0.3792 (4)	0.53025 (13)	0.5296 (3)	0.0216 (11)	
H40	0.457018	0.531125	0.530449	0.026*	
C41	0.3264 (4)	0.63622 (12)	0.5696 (3)	0.0173 (10)	
C42	0.2880 (4)	0.67210 (13)	0.5430 (3)	0.0237 (11)	
H42	0.276531	0.680940	0.476120	0.028*	
C43	0.2662 (5)	0.69537 (14)	0.6159 (4)	0.0348 (13)	
H43	0.240436	0.719544	0.597655	0.042*	
C44	0.2831 (4)	0.68214 (15)	0.7138 (4)	0.0334 (13)	
H44	0.269937	0.697685	0.762477	0.040*	
C45	0.3193 (4)	0.64629 (15)	0.7419 (3)	0.0295 (12)	
H45	0.328276	0.637497	0.808180	0.035*	
C46	0.3421 (4)	0.62350 (13)	0.6706 (3)	0.0203 (10)	
H46	0.368134	0.599408	0.689833	0.024*	
C47	0.1854 (4)	0.54327 (12)	0.2099 (3)	0.0190 (10)	
C48	0.0931 (4)	0.52009 (13)	0.1484 (3)	0.0258 (11)	
H48	0.018084	0.530064	0.109826	0.031*	
C49	0.1112 (5)	0.48275 (14)	0.1440 (4)	0.0328 (13)	
H49	0.047654	0.467587	0.104805	0.039*	
C50	0.2232 (5)	0.46751 (15)	0.1974 (4)	0.0367 (14)	
H50	0.234927	0.442181	0.194608	0.044*	
C51	0.3172 (5)	0.49003 (15)	0.2546 (4)	0.0359 (13)	
H51	0.393402	0.480116	0.288828	0.043*	
C52	0.2979 (4)	0.52767 (13)	0.2611 (3)	0.0227 (11)	
H52	0.361714	0.542713	0.300490	0.027*	
C53	0.0484 (4)	0.60009 (13)	0.2734 (3)	0.0186 (10)	
C54	0.0450 (4)	0.63486 (13)	0.3149 (3)	0.0231 (11)	
H54	0.102310	0.652383	0.316344	0.028*	
C55	−0.0423 (4)	0.64388 (14)	0.3541 (3)	0.0281 (12)	
H55	−0.043485	0.667238	0.382068	0.034*	
C56	−0.1277 (4)	0.61781 (14)	0.3515 (3)	0.0294 (12)	
H56	−0.185895	0.623613	0.378580	0.035*	
C57	−0.1272 (4)	0.58350 (14)	0.3091 (3)	0.0256 (12)	
H57	−0.186297	0.566375	0.305649	0.031*	
C58	−0.0381 (4)	0.57431 (14)	0.2713 (3)	0.0235 (11)	
H58	−0.036596	0.550782	0.244489	0.028*	
C59	0.0822 (4)	0.60429 (12)	0.0805 (3)	0.0188 (10)	
C60	−0.0369 (4)	0.61515 (12)	0.0426 (3)	0.0223 (11)	
H60	−0.077921	0.616660	0.088156	0.027*	
C61	−0.0950 (4)	0.62378 (14)	−0.0626 (3)	0.0298 (12)	
H61	−0.175186	0.630961	−0.087322	0.036*	
C62	−0.0358 (5)	0.62189 (14)	−0.1314 (3)	0.0312 (12)	
H62	−0.074977	0.628004	−0.201958	0.037*	
C63	0.0821 (5)	0.61082 (14)	−0.0938 (3)	0.0299 (12)	
H63	0.122595	0.609225	−0.139794	0.036*	
C64	0.1415 (4)	0.60201 (13)	0.0108 (3)	0.0244 (11)	
H64	0.221358	0.594538	0.034901	0.029*	

### Atomic displacement parameters (Å^2^)

**Table:**

	*U*^11^	*U*^22^	*U*^33^	*U*^12^	*U*^13^	*U*^23^
Pt1	0.01128 (9)	0.01589 (10)	0.01397 (8)	−0.00082 (8)	0.00420 (7)	0.00141 (7)
Fe1	0.0129 (3)	0.0159 (4)	0.0171 (3)	−0.0014 (3)	0.0053 (3)	0.0025 (3)
S1	0.0380 (8)	0.0434 (9)	0.0191 (6)	0.0099 (7)	0.0109 (6)	0.0031 (6)
S2	0.0228 (7)	0.0292 (7)	0.0210 (6)	0.0072 (6)	0.0087 (5)	0.0055 (5)
P1	0.0125 (6)	0.0177 (7)	0.0162 (5)	−0.0015 (5)	0.0035 (5)	0.0004 (5)
P2	0.0119 (6)	0.0134 (6)	0.0149 (5)	0.0001 (5)	0.0043 (5)	0.0015 (5)
P3	0.0129 (6)	0.0200 (7)	0.0159 (5)	−0.0013 (5)	0.0043 (5)	−0.0010 (5)
O1	0.026 (2)	0.026 (2)	0.038 (2)	0.0081 (17)	0.0105 (17)	−0.0009 (16)
O2	0.017 (2)	0.032 (2)	0.051 (2)	−0.0051 (17)	0.0128 (17)	0.0101 (17)
O3	0.0226 (19)	0.0190 (19)	0.0261 (17)	0.0048 (15)	0.0091 (15)	0.0030 (14)
C1	0.018 (3)	0.015 (3)	0.016 (2)	−0.004 (2)	0.0038 (19)	0.0017 (18)
C2	0.021 (3)	0.017 (3)	0.018 (2)	−0.003 (2)	0.009 (2)	−0.0023 (19)
C3	0.012 (2)	0.027 (3)	0.022 (2)	−0.003 (2)	0.008 (2)	−0.003 (2)
C4	0.023 (3)	0.019 (3)	0.022 (2)	−0.001 (2)	0.008 (2)	0.000 (2)
C5	0.028 (3)	0.036 (3)	0.018 (2)	−0.001 (2)	0.006 (2)	−0.009 (2)
C6	0.036 (3)	0.046 (4)	0.029 (3)	−0.002 (3)	0.016 (3)	−0.005 (3)
C7	0.063 (4)	0.029 (3)	0.030 (3)	−0.006 (3)	0.016 (3)	−0.002 (2)
C8	0.045 (4)	0.032 (4)	0.032 (3)	0.002 (3)	0.002 (3)	−0.010 (3)
C9	0.036 (4)	0.045 (4)	0.050 (3)	0.006 (3)	0.016 (3)	−0.013 (3)
C10	0.034 (3)	0.044 (4)	0.034 (3)	−0.007 (3)	0.017 (3)	−0.009 (3)
C11	0.082 (5)	0.040 (4)	0.062 (4)	0.020 (4)	0.015 (4)	−0.003 (3)
C12	0.014 (3)	0.026 (3)	0.023 (2)	−0.002 (2)	0.008 (2)	0.003 (2)
C13	0.019 (3)	0.024 (3)	0.019 (2)	0.001 (2)	0.004 (2)	0.002 (2)
C14	0.016 (3)	0.036 (3)	0.023 (2)	0.004 (2)	0.005 (2)	0.007 (2)
C15	0.026 (3)	0.042 (3)	0.023 (3)	0.001 (3)	0.011 (2)	0.005 (2)
C16	0.026 (3)	0.027 (3)	0.025 (3)	0.002 (2)	0.007 (2)	0.000 (2)
C17	0.015 (3)	0.033 (3)	0.032 (3)	0.003 (2)	0.004 (2)	0.001 (2)
C18	0.019 (3)	0.031 (3)	0.026 (3)	0.002 (2)	0.010 (2)	0.004 (2)
C19	0.035 (3)	0.054 (4)	0.034 (3)	0.004 (3)	0.006 (3)	0.011 (3)
C20	0.020 (3)	0.022 (3)	0.026 (2)	0.005 (2)	0.007 (2)	0.004 (2)
C21	0.016 (3)	0.021 (3)	0.021 (2)	−0.003 (2)	0.006 (2)	0.003 (2)
C22	0.009 (2)	0.027 (3)	0.015 (2)	0.002 (2)	0.0027 (18)	0.0000 (19)
C23	0.020 (3)	0.033 (3)	0.020 (2)	−0.001 (2)	0.006 (2)	0.000 (2)
C24	0.017 (3)	0.056 (4)	0.022 (3)	−0.014 (3)	0.006 (2)	−0.002 (3)
C25	0.010 (3)	0.066 (4)	0.021 (2)	0.002 (3)	0.005 (2)	−0.006 (3)
C26	0.020 (3)	0.040 (3)	0.030 (3)	0.011 (3)	0.005 (2)	−0.011 (2)
C27	0.021 (3)	0.028 (3)	0.019 (2)	0.005 (2)	0.005 (2)	−0.003 (2)
C28	0.013 (2)	0.019 (3)	0.023 (2)	0.000 (2)	0.002 (2)	−0.004 (2)
C29	0.035 (3)	0.024 (3)	0.026 (3)	−0.001 (2)	−0.003 (2)	−0.006 (2)
C30	0.041 (4)	0.025 (3)	0.046 (3)	0.006 (3)	0.001 (3)	−0.011 (3)
C31	0.027 (3)	0.042 (4)	0.038 (3)	0.000 (3)	0.012 (2)	−0.021 (3)
C32	0.032 (3)	0.043 (4)	0.031 (3)	−0.011 (3)	0.016 (2)	−0.010 (3)
C33	0.030 (3)	0.018 (3)	0.028 (3)	−0.001 (2)	0.011 (2)	−0.003 (2)
C34	0.015 (2)	0.016 (3)	0.012 (2)	0.002 (2)	0.0037 (18)	0.0012 (18)
C35	0.019 (3)	0.017 (3)	0.010 (2)	−0.002 (2)	0.0045 (19)	0.0010 (18)
C36	0.021 (3)	0.021 (3)	0.020 (2)	−0.002 (2)	0.004 (2)	0.000 (2)
C37	0.028 (3)	0.032 (3)	0.029 (3)	−0.013 (3)	0.013 (2)	−0.006 (2)
C38	0.048 (4)	0.019 (3)	0.034 (3)	−0.017 (3)	0.018 (3)	−0.001 (2)
C39	0.038 (3)	0.016 (3)	0.029 (3)	0.005 (2)	0.010 (2)	0.000 (2)
C40	0.021 (3)	0.022 (3)	0.022 (2)	0.000 (2)	0.008 (2)	0.003 (2)
C41	0.011 (2)	0.018 (3)	0.023 (2)	−0.005 (2)	0.0069 (19)	−0.0025 (19)
C42	0.027 (3)	0.021 (3)	0.027 (2)	0.000 (2)	0.015 (2)	−0.001 (2)
C43	0.039 (3)	0.020 (3)	0.052 (3)	0.003 (3)	0.025 (3)	−0.006 (3)
C44	0.026 (3)	0.044 (4)	0.036 (3)	−0.009 (3)	0.017 (2)	−0.022 (3)
C45	0.025 (3)	0.044 (4)	0.021 (2)	−0.003 (3)	0.010 (2)	−0.004 (2)
C46	0.015 (2)	0.022 (3)	0.023 (2)	−0.004 (2)	0.0062 (19)	−0.001 (2)
C47	0.022 (3)	0.019 (3)	0.019 (2)	0.000 (2)	0.010 (2)	0.0020 (19)
C48	0.021 (3)	0.027 (3)	0.027 (3)	−0.004 (2)	0.006 (2)	−0.004 (2)
C49	0.037 (3)	0.027 (3)	0.030 (3)	−0.012 (3)	0.008 (3)	−0.007 (2)
C50	0.057 (4)	0.021 (3)	0.031 (3)	0.001 (3)	0.016 (3)	0.000 (2)
C51	0.035 (3)	0.033 (3)	0.035 (3)	0.008 (3)	0.008 (3)	0.004 (3)
C52	0.026 (3)	0.017 (3)	0.022 (2)	−0.004 (2)	0.005 (2)	−0.005 (2)
C53	0.013 (3)	0.027 (3)	0.014 (2)	−0.002 (2)	0.0023 (19)	0.0020 (19)
C54	0.016 (3)	0.028 (3)	0.023 (2)	0.001 (2)	0.005 (2)	0.003 (2)
C55	0.026 (3)	0.032 (3)	0.029 (3)	0.009 (3)	0.014 (2)	0.001 (2)
C56	0.020 (3)	0.046 (4)	0.025 (2)	0.006 (3)	0.011 (2)	0.005 (2)
C57	0.014 (3)	0.036 (3)	0.025 (2)	0.000 (2)	0.006 (2)	0.010 (2)
C58	0.017 (3)	0.033 (3)	0.020 (2)	−0.004 (2)	0.005 (2)	0.002 (2)
C59	0.016 (3)	0.019 (3)	0.018 (2)	−0.003 (2)	0.0011 (19)	−0.0021 (19)
C60	0.018 (3)	0.027 (3)	0.022 (2)	0.006 (2)	0.008 (2)	0.007 (2)
C61	0.020 (3)	0.036 (3)	0.024 (2)	0.007 (2)	−0.002 (2)	−0.001 (2)
C62	0.034 (3)	0.037 (3)	0.015 (2)	0.001 (3)	0.001 (2)	0.001 (2)
C63	0.036 (3)	0.040 (3)	0.017 (2)	0.001 (3)	0.014 (2)	−0.001 (2)
C64	0.016 (3)	0.034 (3)	0.022 (2)	0.001 (2)	0.005 (2)	−0.002 (2)

### Geometric parameters (Å, º)

**Table:**

Pt1—Fe1	2.5697 (6)	C26—C27	1.382 (6)
Pt1—P2	2.2850 (10)	C27—H27	0.9300
Pt1—P3	2.2714 (12)	C28—C29	1.383 (6)
Pt1—C1	2.045 (4)	C28—C33	1.385 (6)
Fe1—P1	2.1966 (12)	C29—H29	0.9300
Fe1—C1	2.162 (4)	C29—C30	1.383 (6)
Fe1—C2	2.119 (4)	C30—H30	0.9300
Fe1—C3	1.932 (5)	C30—C31	1.379 (7)
Fe1—C20	1.777 (5)	C31—H31	0.9300
Fe1—C21	1.789 (5)	C31—C32	1.356 (7)
S1—C4	1.830 (4)	C32—H32	0.9300
S1—C5	1.782 (5)	C32—C33	1.378 (6)
S2—C12	1.808 (4)	C33—H33	0.9300
S2—C13	1.771 (4)	C34—H34A	0.9700
P1—C22	1.839 (4)	C34—H34B	0.9700
P1—C28	1.841 (4)	C35—C36	1.399 (6)
P1—C34	1.842 (4)	C35—C40	1.385 (6)
P2—C34	1.829 (4)	C36—H36	0.9300
P2—C35	1.824 (4)	C36—C37	1.378 (6)
P2—C41	1.819 (4)	C37—H37	0.9300
P3—C47	1.824 (5)	C37—C38	1.388 (7)
P3—C53	1.824 (4)	C38—H38	0.9300
P3—C59	1.830 (4)	C38—C39	1.376 (7)
O1—C21	1.157 (5)	C39—H39	0.9300
O2—C20	1.141 (5)	C39—C40	1.377 (6)
O3—C3	1.216 (5)	C40—H40	0.9300
C1—C2	1.407 (6)	C41—C42	1.380 (6)
C1—C12	1.483 (6)	C41—C46	1.402 (6)
C2—C3	1.464 (6)	C42—H42	0.9300
C2—C4	1.511 (5)	C42—C43	1.404 (6)
C4—H4A	0.9700	C43—H43	0.9300
C4—H4B	0.9700	C43—C44	1.366 (6)
C5—C6	1.384 (6)	C44—H44	0.9300
C5—C10	1.397 (6)	C44—C45	1.376 (7)
C6—H6	0.9300	C45—H45	0.9300
C6—C7	1.367 (7)	C45—C46	1.381 (6)
C7—H7	0.9300	C46—H46	0.9300
C7—C8	1.392 (7)	C47—C48	1.394 (6)
C8—C9	1.373 (7)	C47—C52	1.388 (6)
C8—C11	1.513 (7)	C48—H48	0.9300
C9—H9	0.9300	C48—C49	1.373 (7)
C9—C10	1.381 (7)	C49—H49	0.9300
C10—H10	0.9300	C49—C50	1.382 (7)
C11—H11A	0.9600	C50—H50	0.9300
C11—H11B	0.9600	C50—C51	1.374 (7)
C11—H11C	0.9600	C51—H51	0.9300
C12—H12A	0.9700	C51—C52	1.389 (6)
C12—H12B	0.9700	C52—H52	0.9300
C13—C14	1.394 (6)	C53—C54	1.387 (6)
C13—C18	1.390 (6)	C53—C58	1.389 (6)
C14—H14	0.9300	C54—H54	0.9300
C14—C15	1.387 (6)	C54—C55	1.384 (6)
C15—H15	0.9300	C55—H55	0.9300
C15—C16	1.378 (6)	C55—C56	1.383 (7)
C16—C17	1.395 (6)	C56—H56	0.9300
C16—C19	1.516 (6)	C56—C57	1.371 (7)
C17—H17	0.9300	C57—H57	0.9300
C17—C18	1.373 (6)	C57—C58	1.391 (6)
C18—H18	0.9300	C58—H58	0.9300
C19—H19A	0.9600	C59—C60	1.383 (6)
C19—H19B	0.9600	C59—C64	1.390 (6)
C19—H19C	0.9600	C60—H60	0.9300
C22—C23	1.404 (6)	C60—C61	1.382 (6)
C22—C27	1.376 (6)	C61—H61	0.9300
C23—H23	0.9300	C61—C62	1.378 (6)
C23—C24	1.381 (6)	C62—H62	0.9300
C24—H24	0.9300	C62—C63	1.372 (7)
C24—C25	1.377 (7)	C63—H63	0.9300
C25—H25	0.9300	C63—C64	1.379 (6)
C25—C26	1.388 (7)	C64—H64	0.9300
C26—H26	0.9300		
			
P2—Pt1—Fe1	97.26 (3)	C24—C23—C22	120.3 (5)
P3—Pt1—Fe1	161.46 (3)	C24—C23—H23	119.9
P3—Pt1—P2	100.53 (4)	C23—C24—H24	119.7
C1—Pt1—Fe1	54.44 (12)	C25—C24—C23	120.5 (5)
C1—Pt1—P2	151.36 (12)	C25—C24—H24	119.7
C1—Pt1—P3	107.33 (12)	C24—C25—H25	120.2
P1—Fe1—Pt1	93.50 (4)	C24—C25—C26	119.5 (5)
C1—Fe1—Pt1	50.30 (11)	C26—C25—H25	120.2
C1—Fe1—P1	141.85 (12)	C25—C26—H26	120.0
C2—Fe1—Pt1	73.72 (12)	C27—C26—C25	119.9 (5)
C2—Fe1—P1	130.91 (13)	C27—C26—H26	120.0
C2—Fe1—C1	38.35 (16)	C22—C27—C26	121.3 (5)
C3—Fe1—Pt1	72.18 (13)	C22—C27—H27	119.3
C3—Fe1—P1	88.88 (13)	C26—C27—H27	119.3
C3—Fe1—C1	70.43 (18)	C29—C28—P1	120.7 (3)
C3—Fe1—C2	42.03 (17)	C29—C28—C33	118.0 (4)
C20—Fe1—Pt1	168.78 (14)	C33—C28—P1	121.2 (4)
C20—Fe1—P1	95.66 (14)	C28—C29—H29	119.5
C20—Fe1—C1	119.19 (18)	C28—C29—C30	120.9 (5)
C20—Fe1—C2	95.33 (18)	C30—C29—H29	119.5
C20—Fe1—C3	101.5 (2)	C29—C30—H30	120.1
C20—Fe1—C21	96.5 (2)	C31—C30—C29	119.8 (5)
C21—Fe1—Pt1	87.76 (14)	C31—C30—H30	120.1
C21—Fe1—P1	102.63 (13)	C30—C31—H31	120.1
C21—Fe1—C1	89.16 (18)	C32—C31—C30	119.8 (5)
C21—Fe1—C2	123.33 (18)	C32—C31—H31	120.1
C21—Fe1—C3	157.6 (2)	C31—C32—H32	119.6
C5—S1—C4	102.1 (2)	C31—C32—C33	120.7 (5)
C13—S2—C12	102.2 (2)	C33—C32—H32	119.6
C22—P1—Fe1	115.03 (13)	C28—C33—H33	119.6
C22—P1—C28	100.05 (19)	C32—C33—C28	120.7 (5)
C22—P1—C34	102.6 (2)	C32—C33—H33	119.6
C28—P1—Fe1	121.17 (15)	P1—C34—H34A	109.6
C28—P1—C34	103.71 (19)	P1—C34—H34B	109.6
C34—P1—Fe1	112.02 (13)	P2—C34—P1	110.3 (2)
C34—P2—Pt1	103.14 (13)	P2—C34—H34A	109.6
C35—P2—Pt1	121.79 (13)	P2—C34—H34B	109.6
C35—P2—C34	102.5 (2)	H34A—C34—H34B	108.1
C41—P2—Pt1	121.93 (15)	C36—C35—P2	118.3 (3)
C41—P2—C34	103.99 (19)	C40—C35—P2	123.3 (3)
C41—P2—C35	100.66 (19)	C40—C35—C36	118.5 (4)
C47—P3—Pt1	114.72 (15)	C35—C36—H36	119.9
C47—P3—C53	108.4 (2)	C37—C36—C35	120.2 (5)
C47—P3—C59	100.6 (2)	C37—C36—H36	119.9
C53—P3—Pt1	111.32 (15)	C36—C37—H37	119.8
C53—P3—C59	101.2 (2)	C36—C37—C38	120.4 (5)
C59—P3—Pt1	119.15 (15)	C38—C37—H37	119.8
Pt1—C1—Fe1	75.25 (13)	C37—C38—H38	120.2
C2—C1—Pt1	109.1 (3)	C39—C38—C37	119.6 (5)
C2—C1—Fe1	69.2 (2)	C39—C38—H38	120.2
C2—C1—C12	119.6 (4)	C38—C39—H39	120.0
C12—C1—Pt1	130.5 (3)	C38—C39—C40	120.1 (5)
C12—C1—Fe1	129.0 (3)	C40—C39—H39	120.0
C1—C2—Fe1	72.5 (2)	C35—C40—H40	119.4
C1—C2—C3	111.2 (4)	C39—C40—C35	121.2 (4)
C1—C2—C4	126.2 (4)	C39—C40—H40	119.4
C3—C2—Fe1	62.1 (2)	C42—C41—P2	121.3 (3)
C3—C2—C4	121.9 (4)	C42—C41—C46	118.5 (4)
C4—C2—Fe1	124.8 (3)	C46—C41—P2	120.1 (3)
O3—C3—Fe1	146.8 (3)	C41—C42—H42	119.7
O3—C3—C2	136.5 (4)	C41—C42—C43	120.6 (4)
C2—C3—Fe1	75.8 (3)	C43—C42—H42	119.7
S1—C4—H4A	109.0	C42—C43—H43	120.3
S1—C4—H4B	109.0	C44—C43—C42	119.3 (5)
C2—C4—S1	112.9 (3)	C44—C43—H43	120.3
C2—C4—H4A	109.0	C43—C44—H44	119.3
C2—C4—H4B	109.0	C43—C44—C45	121.3 (4)
H4A—C4—H4B	107.8	C45—C44—H44	119.3
C6—C5—S1	121.9 (4)	C44—C45—H45	120.3
C6—C5—C10	118.4 (5)	C44—C45—C46	119.4 (4)
C10—C5—S1	119.7 (4)	C46—C45—H45	120.3
C5—C6—H6	119.8	C41—C46—H46	119.6
C7—C6—C5	120.4 (5)	C45—C46—C41	120.8 (4)
C7—C6—H6	119.8	C45—C46—H46	119.6
C6—C7—H7	118.9	C48—C47—P3	122.3 (4)
C6—C7—C8	122.2 (5)	C52—C47—P3	120.0 (3)
C8—C7—H7	118.9	C52—C47—C48	117.7 (4)
C7—C8—C11	121.0 (5)	C47—C48—H48	119.5
C9—C8—C7	116.9 (5)	C49—C48—C47	121.0 (5)
C9—C8—C11	122.1 (5)	C49—C48—H48	119.5
C8—C9—H9	118.9	C48—C49—H49	119.8
C8—C9—C10	122.2 (5)	C48—C49—C50	120.5 (5)
C10—C9—H9	118.9	C50—C49—H49	119.8
C5—C10—H10	120.0	C49—C50—H50	120.2
C9—C10—C5	119.9 (5)	C51—C50—C49	119.6 (5)
C9—C10—H10	120.0	C51—C50—H50	120.2
C8—C11—H11A	109.5	C50—C51—H51	120.1
C8—C11—H11B	109.5	C50—C51—C52	119.8 (5)
C8—C11—H11C	109.5	C52—C51—H51	120.1
H11A—C11—H11B	109.5	C47—C52—C51	121.3 (4)
H11A—C11—H11C	109.5	C47—C52—H52	119.3
H11B—C11—H11C	109.5	C51—C52—H52	119.3
S2—C12—H12A	109.1	C54—C53—P3	116.1 (3)
S2—C12—H12B	109.1	C54—C53—C58	118.7 (4)
C1—C12—S2	112.3 (3)	C58—C53—P3	125.1 (4)
C1—C12—H12A	109.1	C53—C54—H54	119.5
C1—C12—H12B	109.1	C55—C54—C53	121.0 (5)
H12A—C12—H12B	107.9	C55—C54—H54	119.5
C14—C13—S2	124.4 (4)	C54—C55—H55	120.3
C18—C13—S2	118.1 (3)	C56—C55—C54	119.4 (5)
C18—C13—C14	117.5 (4)	C56—C55—H55	120.3
C13—C14—H14	119.6	C55—C56—H56	119.8
C15—C14—C13	120.7 (4)	C57—C56—C55	120.5 (4)
C15—C14—H14	119.6	C57—C56—H56	119.8
C14—C15—H15	119.1	C56—C57—H57	120.0
C16—C15—C14	121.7 (4)	C56—C57—C58	120.0 (5)
C16—C15—H15	119.1	C58—C57—H57	120.0
C15—C16—C17	117.2 (4)	C53—C58—C57	120.4 (5)
C15—C16—C19	121.7 (4)	C53—C58—H58	119.8
C17—C16—C19	121.1 (5)	C57—C58—H58	119.8
C16—C17—H17	119.2	C60—C59—P3	122.2 (3)
C18—C17—C16	121.6 (5)	C60—C59—C64	118.9 (4)
C18—C17—H17	119.2	C64—C59—P3	118.9 (3)
C13—C18—H18	119.4	C59—C60—H60	119.9
C17—C18—C13	121.2 (4)	C61—C60—C59	120.2 (4)
C17—C18—H18	119.4	C61—C60—H60	119.9
C16—C19—H19A	109.5	C60—C61—H61	119.6
C16—C19—H19B	109.5	C62—C61—C60	120.9 (5)
C16—C19—H19C	109.5	C62—C61—H61	119.6
H19A—C19—H19B	109.5	C61—C62—H62	120.6
H19A—C19—H19C	109.5	C63—C62—C61	118.8 (4)
H19B—C19—H19C	109.5	C63—C62—H62	120.6
O2—C20—Fe1	178.7 (4)	C62—C63—H63	119.4
O1—C21—Fe1	179.9 (5)	C62—C63—C64	121.1 (4)
C23—C22—P1	116.6 (4)	C64—C63—H63	119.4
C27—C22—P1	124.9 (4)	C59—C64—H64	119.9
C27—C22—C23	118.4 (4)	C63—C64—C59	120.1 (4)
C22—C23—H23	119.9	C63—C64—H64	119.9
			
Pt1—P2—C34—P1	52.0 (2)	C22—P1—C28—C29	109.4 (4)
Pt1—P2—C35—C36	−87.5 (3)	C22—P1—C28—C33	−70.4 (4)
Pt1—P2—C35—C40	92.7 (4)	C22—P1—C34—P2	−172.0 (2)
Pt1—P2—C41—C42	−5.3 (4)	C22—C23—C24—C25	0.1 (6)
Pt1—P2—C41—C46	177.8 (3)	C23—C22—C27—C26	−0.6 (6)
Pt1—P3—C47—C48	−174.1 (3)	C23—C24—C25—C26	−1.6 (7)
Pt1—P3—C47—C52	2.6 (4)	C24—C25—C26—C27	2.0 (7)
Pt1—P3—C53—C54	34.5 (4)	C25—C26—C27—C22	−0.9 (7)
Pt1—P3—C53—C58	−148.9 (3)	C27—C22—C23—C24	1.0 (6)
Pt1—P3—C59—C60	−122.1 (4)	C28—P1—C22—C23	−58.9 (3)
Pt1—P3—C59—C64	57.8 (4)	C28—P1—C22—C27	124.9 (4)
Pt1—C1—C2—Fe1	−65.2 (2)	C28—P1—C34—P2	84.2 (2)
Pt1—C1—C2—C3	−15.6 (4)	C28—C29—C30—C31	0.1 (8)
Pt1—C1—C2—C4	174.2 (3)	C29—C28—C33—C32	1.8 (7)
Pt1—C1—C12—S2	18.9 (5)	C29—C30—C31—C32	1.4 (8)
Fe1—P1—C22—C23	72.6 (3)	C30—C31—C32—C33	−1.4 (8)
Fe1—P1—C22—C27	−103.6 (3)	C31—C32—C33—C28	−0.2 (8)
Fe1—P1—C28—C29	−18.2 (5)	C33—C28—C29—C30	−1.7 (7)
Fe1—P1—C28—C33	162.1 (3)	C34—P1—C22—C23	−165.5 (3)
Fe1—P1—C34—P2	−48.1 (2)	C34—P1—C22—C27	18.3 (4)
Fe1—C1—C2—C3	49.6 (3)	C34—P1—C28—C29	−144.9 (4)
Fe1—C1—C2—C4	−120.6 (4)	C34—P1—C28—C33	35.3 (4)
Fe1—C1—C12—S2	−86.2 (4)	C34—P2—C35—C36	158.2 (3)
Fe1—C2—C3—O3	−170.8 (6)	C34—P2—C35—C40	−21.6 (4)
Fe1—C2—C4—S1	−172.3 (2)	C34—P2—C41—C42	110.2 (4)
S1—C5—C6—C7	−179.4 (4)	C34—P2—C41—C46	−66.6 (4)
S1—C5—C10—C9	180.0 (4)	C35—P2—C34—P1	179.29 (19)
S2—C13—C14—C15	−175.3 (4)	C35—P2—C41—C42	−143.8 (4)
S2—C13—C18—C17	176.1 (4)	C35—P2—C41—C46	39.3 (4)
P1—C22—C23—C24	−175.4 (3)	C35—C36—C37—C38	0.6 (6)
P1—C22—C27—C26	175.5 (3)	C36—C35—C40—C39	−0.8 (6)
P1—C28—C29—C30	178.5 (4)	C36—C37—C38—C39	−0.1 (7)
P1—C28—C33—C32	−178.5 (4)	C37—C38—C39—C40	−0.8 (7)
P2—C35—C36—C37	−179.9 (3)	C38—C39—C40—C35	1.3 (7)
P2—C35—C40—C39	179.0 (3)	C40—C35—C36—C37	−0.1 (6)
P2—C41—C42—C43	−176.3 (4)	C41—P2—C34—P1	−76.2 (2)
P2—C41—C46—C45	177.1 (3)	C41—P2—C35—C36	51.1 (3)
P3—C47—C48—C49	−179.2 (3)	C41—P2—C35—C40	−128.7 (4)
P3—C47—C52—C51	−179.3 (3)	C41—C42—C43—C44	−0.1 (7)
P3—C53—C54—C55	177.3 (3)	C42—C41—C46—C45	0.2 (7)
P3—C53—C58—C57	−175.9 (3)	C42—C43—C44—C45	−1.1 (8)
P3—C59—C60—C61	179.5 (4)	C43—C44—C45—C46	1.8 (7)
P3—C59—C64—C63	−179.3 (4)	C44—C45—C46—C41	−1.3 (7)
C1—C2—C3—Fe1	−55.2 (3)	C46—C41—C42—C43	0.5 (7)
C1—C2—C3—O3	134.0 (6)	C47—P3—C53—C54	161.5 (3)
C1—C2—C4—S1	−79.3 (5)	C47—P3—C53—C58	−21.8 (4)
C2—C1—C12—S2	−172.5 (3)	C47—P3—C59—C60	111.6 (4)
C3—C2—C4—S1	111.4 (4)	C47—P3—C59—C64	−68.5 (4)
C4—S1—C5—C6	72.5 (4)	C47—C48—C49—C50	−2.6 (7)
C4—S1—C5—C10	−108.5 (4)	C48—C47—C52—C51	−2.3 (6)
C4—C2—C3—Fe1	115.5 (4)	C48—C49—C50—C51	−0.5 (7)
C4—C2—C3—O3	−55.3 (8)	C49—C50—C51—C52	2.1 (7)
C5—S1—C4—C2	−78.1 (4)	C50—C51—C52—C47	−0.6 (7)
C5—C6—C7—C8	−1.0 (8)	C52—C47—C48—C49	3.9 (6)
C6—C5—C10—C9	−0.9 (7)	C53—P3—C47—C48	60.8 (4)
C6—C7—C8—C9	−0.2 (8)	C53—P3—C47—C52	−122.4 (3)
C6—C7—C8—C11	179.4 (5)	C53—P3—C59—C60	0.3 (4)
C7—C8—C9—C10	0.8 (8)	C53—P3—C59—C64	−179.9 (4)
C8—C9—C10—C5	−0.3 (8)	C53—C54—C55—C56	−0.3 (7)
C10—C5—C6—C7	1.6 (7)	C54—C53—C58—C57	0.7 (6)
C11—C8—C9—C10	−178.7 (5)	C54—C55—C56—C57	−0.9 (7)
C12—S2—C13—C14	−36.3 (5)	C55—C56—C57—C58	1.9 (7)
C12—S2—C13—C18	146.7 (4)	C56—C57—C58—C53	−1.9 (7)
C12—C1—C2—Fe1	124.0 (4)	C58—C53—C54—C55	0.4 (6)
C12—C1—C2—C3	173.6 (4)	C59—P3—C47—C48	−44.9 (4)
C12—C1—C2—C4	3.3 (7)	C59—P3—C47—C52	131.8 (3)
C13—S2—C12—C1	−166.4 (3)	C59—P3—C53—C54	−93.2 (3)
C13—C14—C15—C16	−1.5 (8)	C59—P3—C53—C58	83.5 (4)
C14—C13—C18—C17	−1.1 (7)	C59—C60—C61—C62	−0.3 (8)
C14—C15—C16—C17	0.6 (7)	C60—C59—C64—C63	0.6 (7)
C14—C15—C16—C19	179.8 (5)	C60—C61—C62—C63	0.8 (8)
C15—C16—C17—C18	−0.1 (7)	C61—C62—C63—C64	−0.6 (8)
C16—C17—C18—C13	0.4 (8)	C62—C63—C64—C59	−0.1 (8)
C18—C13—C14—C15	1.7 (7)	C64—C59—C60—C61	−0.4 (7)
C19—C16—C17—C18	−179.3 (5)		

### Hydrogen-bond geometry (Å, º)

**Table:**

*D*—H···*A*	*D*—H	H···*A*	*D*···*A*	*D*—H···*A*
C15—H15···O1^i^	0.93	2.67	3.316 (6)	128
C34—H34*A*···O3	0.97	2.62	3.271 (5)	125
C39—H39···O3^ii^	0.93	2.49	3.239 (6)	138

Symmetry codes: (i) *x*, −*y*+3/2, *z*−1/2; (ii) −*x*+1, −*y*+1, −*z*+1.
